# Treating fatty liver and insulin resistance

**DOI:** 10.18632/aging.100617

**Published:** 2013-11-30

**Authors:** Rachel J. Perry, Gerald I. Shulman

**Affiliations:** ^1^ Howard Hughes Medical Institute, Yale University School of Medicine, New Haven, CT 06519, USA; ^2^ Department of Internal Medicine, Yale University School of Medicine, New Haven, CT 06519, USA; ^3^ Department of Cellular & Molecular Physiology, Yale University School of Medicine, New Haven, CT 06519, USA New Haven, CT 06519, USA; ^4^ Novo Nordisk Foundation Center for Basic Biomedical Research, Copenhagen, DK

Type 2 diabetes (T2D) is predicted to affect one in three Americans by the year 2050 and is a leading cause of blindness, end stage renal failure and loss of limb with associated costs exceeding $180 billion/year. Insulin resistance in liver and skeletal muscle are major factors in the pathogenesis of T2D, with a strong relationship between ectopic lipid content in liver and skeletal muscle with insulin resistance and T2D in both humans and rodent models of NAFLD and T2D (Shulman, J. Clin. Invest. 2000; 106:171-6, Samuel and Shulman, Cell 2012; 148:852-71).

Evidence in support of the key role of ectopic lipid in the pathogenesis of hepatic insulin resistance and T2D in humans comes from studies where we (Petersen et al., Diabetes 2005; 54:603-608) and others (Lim et al., Diabetologia 2011, 54:2506-2514) have shown that modest weight loss (~8 kg) resulted in large (~80%) reductions in liver triglyceride content, which were associated with normalization of fasting plasma glucose concentrations, hepatic glucose production and hepatic insulin sensitivity. Thus we hypothesized that reversal of non-alcoholic fatty liver disease (NAFLD), by promoting mild mitochondrial uncoupling would reverse insulin resistance and diabetes in rat models of NAFLD and T2D. Previous studies by our group had found that treating high fat fed rats with 2,4-dinitrophenol (DNP), a well established mitochondrial protonophore, would lead to mitochondrial energy uncoupling leading to reductions in liver triacylglycerol (TAG) and DAG content, decreased PKCε activation and decreased hepatic insulin resistance (Samuel et al., J. Biol. Chem. 2004; 279:32345-53). DNP uncouples mitochondrial glucose and fat oxidation from ATP production by shuttling protons across the mitochondrial membrane resulting in increased mitochondrial energy consumption, but can be fatally toxic due to its on-target side effect of hyperthermia. We hypothesized that we could markedly increase the therapeutic window of DNP by targeting it to the liver. We therefore generated several derivatives of DNP, which would be preferentially metabolized by the cytochrome P-450 system in the liver to the active protonophore, DNP, and screened them in isolated hepatocytes for their ability to promote increased oxygen consumption. From this screen we identified DNP-methyl ether (DNPME), which raised oxygen consumption rates with similar potencies to DNP. As predicted, the ratio of toxic to effective dose was 50-fold greater for DNPME than DNP, without any evidence of hyperthermia or renal or hepatic toxicity in rats treated for 6 weeks with DNPME. This therapeutic index also compares favorably with other drugs that are in common use such as acetaminophen, which has a LD_50_/ED_50_ of 13.

Given the markedly increased therapeutic window for DNPME versus DNP we then examined whether DNPME would reverse insulin resistance, hepatic steatosis and diabetes in the rat. Despite identical body weights at the time of study, DNPME-treated rats had lower fasting plasma glucose, insulin, and triglyceride concentrations; improved glucose tolerance; and increased hepatic and peripheral insulin sensitivity. These improvements in liver and muscle insulin responsiveness were associated with reductions in liver and muscle TAG and DAG content as well as decreases in PKC activity in liver and skeletal muscle. In contrast there were no changes in tissue ceramide content or inflammatory markers with DNPME treatment, disassociating these factors from reversal of insulin resistance and diabetes in these animals.

Given the surprising effect of DNPME to increase peripheral insulin sensitivity we examined the effects of DNPME on hepatic VLDL production and found that DNPME treatment resulted in a ~50% reduction in hepatic VLDL export, consistent with DNPME induced reductions in plasma triglyceride concentrations. In addition we observed that DNPME treatment caused a 60% increase in hepatic tricarboxylic acid (TCA) cycle flux whereas there were no observable effects of DNPME treatment on relative rates of mitochondrial fatty acid/TCA cycle flux in heart, skeletal muscle, brain, or kidney. Taken together these data imply that DNPME treatment promotes increased hepatic mitochondrial oxidation rates resulting in lower hepatic TAG and DAG content and reduced VLDL production resulting in decreased plasma triglyceride concentrations and decreased muscle TAG and DAG content (Figure).

Because DNPME treatment promoted regression of NAFLD and insulin resistance in high fat fed rat models of NAFLD, we next examined whether DNPME would also be effective in two rat models of T2D. DNPME treatment led to a reduction in fasting plasma glucose, insulin, and triglyceride concentrations, improved glucose tolerance and reduced hepatic and peripheral muscle lipid content without any indication of hepatic or renal toxicity in both rat models, highlighting its potential for use as a therapeutic agent in patients with T2D.

In summary these data demonstrate that by promoting a relatively small increase in hepatic mitochondrial uncoupling, DNPME can safely reverse hepatic steatosis, hypertriglyceridemia and insulin resistance in a rat model of NAFLD without inducing hyperthermia or associated systemic toxicities. In addition we also found that DNPME reduces fasting plasma glucose and insulin concentrations and improves glucose tolerance in two rat models of T2D. Taken together these data demonstrate the potential feasibility of disassociating the toxicity of DNP from its efficacy by altering the pharmacokinetics of DNP metabolism to treat the related epidemics of NAFLD, metabolic syndrome and type 2 diabetes.

**Figure 1:**
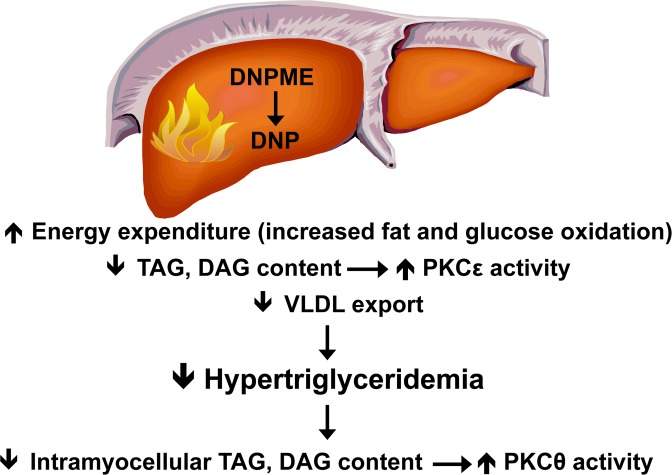
Mechanism by which DNPME reverses liver and muscle insulin resistance.

